# Canavanine Alters ROS/RNS Level and Leads to Post-translational Modification of Proteins in Roots of Tomato Seedlings

**DOI:** 10.3389/fpls.2016.00840

**Published:** 2016-06-14

**Authors:** Urszula Krasuska, Olga Andrzejczak, Paweł Staszek, Renata Bogatek, Agnieszka Gniazdowska

**Affiliations:** Department of Plant Physiology, Faculty of Agriculture and Biology, Warsaw University of Life Sciences-SGGWWarsaw, Poland

**Keywords:** NADPH oxidase, peroxidase, polyamine oxidase, proteolysis, protein carbonylation, protein nitration, RNS, ROS

## Abstract

Canavanine (CAN), a structural analog of arginine (Arg), is used as a selective inhibitor of inducible NOS in mammals. CAN is incorporated into proteins’ structure in the place of Arg, leading to the formation of aberrant compounds. This non-protein amino acid is found in legumes, e.g., *Canavalia ensiformis* (L.) DC. or *Sutherlandia frutescens* (L.) R.Br. and acts as a strong toxin against herbivores or plants. Tomato (*Solanum lycopersicum* L.) seedlings were treated for 24–72 h with CAN (10 or 50 μM) inhibiting root growth by 50 or 100%, without lethal effect. We determined ROS level/production in root extracts, fluorescence of DAF-FM and APF derivatives corresponding to RNS level in roots of tomato seedlings and linked CAN-induced restriction of root growth to the post-translational modifications (PTMs) of proteins: carbonylation and nitration. Both PTMs are stable markers of nitro-oxidative stress, regarded as the plant’s secondary response to phytotoxins. CAN enhanced H_2_O_2_ content and superoxide radicals generation in extracts of tomato roots and stimulated formation of protein carbonyl groups. An elevated level of carbonylated proteins was characteristic for the plants after 72 h of the culture, mainly for the roots exposed to 10 μM CAN. The proteolytic activity was stimulated by tested non-protein amino acid. CAN treatment led to decline of fluorescence of DAF-FM derivatives, and transiently stimulated fluorescence of APF derivatives. Short-term exposure of tomato seedlings to CAN lowered the protein nitration level. Activity of peroxidase, polyamine oxidase and NADPH oxidase, enzymes acting as modulators of H_2_O_2_ concentration and governing root architecture and growth were determined. Activities of all enzymes were stimulated by CAN, but no strict CAN concentration dependence was observed. We conclude, that although CAN treatment led to a decline in the nitric oxide level, PTMs observed in roots of plants exposed to CAN are linked rather to the formation of carbonyl groups than to nitration, and are detected particularly after 24 h. Thus, oxidative stress and oxidative modifications of proteins seems to be of significant importance in the rapid response of plants to CAN.

## Introduction

In addition to 20 amino acids, known to act as building blocks of proteins, living organisms also produce non-proteinogenic amino acids (NPAAs). In plants, about 250 NPAAs have been identified (Vranova et al., 2011), which possess a rich structural diversity and some of them exhibit harmful biological effects both in plants and animals (Rosenthal, 1982, 2001; Bell, 2003). The possible role of NPAAs in plants is protection against predators or pathogens. They act also as an allelopathic weapon against neighboring organisms. Among NPAAs naturally occurring in planta L-canavanine (CAN), the L-2-amino-4-(guanidinoxy) butyric acid is identified and recognized as a compound of high toxicity (Rosenthal, 2001), but of great importance in medicine, where is used as cytotoxic agent against cancer cells in humans (Swaffar et al., 1994, 1995; Vynnytska et al., 2011). In animals the mode of action of CAN depends on the fact that this NPAA is the guanidinoxy structural analog of arginine (Arg) and its presence can lead to a production of CAN-containing proteins, which may disrupt cellular metabolism (Rosenthal and Harper, 1996). CAN affects also regulatory and catalytic reactions of Arg metabolism or uptake. According to the toxicological data, CAN is considered to be “very toxic” (Rodricks, 2007), as is linked with several serious diseases in humans, e.g., systemic lupus erythematosus (SLE), which is characterized by a defect in the immune system. The inflammatory and oxidative modification reactions are the most important events associated with complications of SLE patients (Shah et al., 2014). Recently, hyper-nitration of tyrosine residues of, e.g., histone H1 has been suggested as an etiopathogenesis of SLE and rheumatoid arthritis (Khan et al., 2014).

In mammalian tissue NO synthase (NOS) converts Arg to NO and L-citrulline (Stuehr, 1999). Acting as an antimetabolite of Arg, in animal tissue CAN is commonly used as a selective inhibitor of inducible isoform of NOS (iNOS; Abd El-Gawad and Khalifa, 2001; Li et al., 2001). Application of CAN in animal or human tissue to inhibit NOS activity results in a decrease in NO emission (Luzzi and Marletta, 2005), but also induces an oxidative burst demonstrated by an elevated level of ROS (Demiryürek et al., 1997; Riganti et al., 2003).

In plants, like in animal tissue, NO acts as a signaling molecule. But the pathways of its biosynthesis are still not clarified. Among many metabolic pathways such as polyamines (PAs) or ethylene biosynthesis, in plant cells Arg is the hypothetical substrate for NO formation. There is no doubt that in higher plants NO formation depends on reductive pathways utilizing NO_2_^-^ as a substrate, but the existence of the enzyme of NOS-like activity is controversial (Corpas et al., 2009; review by Gupta et al., 2011; Hancock, 2012; Corpas and Barroso, 2015). Nevertheless, both in animals and plants NO mode of action involves PTMs of proteins including *S*-nitrosylation of cysteine (Cys) and nitration of tyrosine (Tyr) residues (Lozano-Juste et al., 2011). In addition, carbonylation of bovine serum albumin (BSA) has been demonstrated *in vitro* after NO donor application (Krasuska et al., 2014b). Nitration of Tyr is connected with a covalent binding of a nitro (-NO_2_) group to one of the two equivalent *ortho* carbons of this amino acid and leads to the formation of 3-nitrotyrosine (NO_2_Tyr, 3-NT; Chaki et al., 2009). Nitration is associated with an accumulation of peroxynitrite (ONOO^-^), the oxidizing product of reaction of superoxide anion ($O_{2}^{•-}$) and NO in its radical form (Arasimowicz-Jelonek and Floryszak-Wieczorek, 2011; Astier and Lindermayr, 2012).

Disturbances in the metabolism of NO or other RNS are related to an alteration in ROS level leading commonly to the induction of nitro-oxidative stress (Corpas and Barroso, 2013). The formation of carbonylated proteins is due to various types of oxidative modification of amino acids, direct ROS attacks on proline (Pro), Arg, lysine (Lys), asparagine (Asn) and threonine (Thr) residues, the incorporation of reactive carbonyl derivatives into peptides by interaction with Cys, histidine (His) and Lys or the adduction of advanced glycation end products formed by ROS reactions with reducing sugars (Møller et al., 2011; Yan and Forster, 2011). It has been proposed that carbonylation, although is a negative consequence of ROS over-accumulation, serves also as an intracellular metabolic regulatory mechanism.

Treatment of tomato (*Solanum lycopersicum* L.) seedlings with CAN (10 μM) for 24 or 72 h led to 50% inhibition of root growth, while the application of CAN at higher concentration (50 μM) entirely inhibited root elongation, but had no lethal effect, although no recovery was observed after seedlings transfer to Arg or water (Krasuska et al., 2016). CAN treatment did not lead to DNA fragmentation, but resulted in slight enhancement in RNA content, accompanied by 20% enlargement in total protein level. Preliminary data indicated that prolonged culture of tomato seedlings in CAN solution increased accumulation of ROS ($O_{2}^{•-}$ and H_2_O_2_) in tomato root tips detected by tissue staining with NBT or DAB (Krasuska et al., 2016). CAN inhibited also NO emission and transiently enhanced ONOO^-^ production in root tips of tomato seedlings, suggesting its direct impact on NO biosynthetic pathway (Krasuska et al., 2016).

The aim of our work was to show another than incorporation into the protein structure mode of action of CAN. We investigated the modifications in ROS concentration or generation rate, and fluorescence of DAF-FM or APF derivatives after reaction with RNS in roots of tomato seedlings after 24–72 h long exposure to CAN, as our previously published data indicated influence of CAN on RNS content in root tips (Krasuska et al., 2016). Following the treatments, formation of carbonyl groups and 3-NT were studied, to detect stable and reliable markers of nitro-oxidative stress, which could be induced by CAN. Some PTMs, e.g., carbonylation stimulate proteolytic degradation, therefore, we investigated impact of CAN on proteolytic activity. In addition, we linked CAN toxicity with the activity of polyamine oxidase (PAO) because decline of PAs content was observed as a result of CAN treatment (Davis, 1997) and degradation of PAs leads to H_2_O_2_ formation. NADPH oxidase catalyze the apoplastic production of $O_{2}^{•-}$, plant peroxidase Class III (POx) catalyzes oxidoreduction between H_2_O_2_ and various reductants (Hiraga et al., 2001; Kaur et al., 2014). Both enzymes govern also root architecture and growth, together with classical phytohormons and NO (Krasuska and Gniazdowska, 2015) therefore, could play an important role in plant response to CAN.

To summarize, the aim of the study was to find the stable markers of CAN toxicity and describe the mode of action of this non-protein amino acid, to show that application of commonly used inhibitor of NO biosynthesis may result in oxidative modifications.

## Materials and Methods

### Plant Material

**FIGURE 1:**
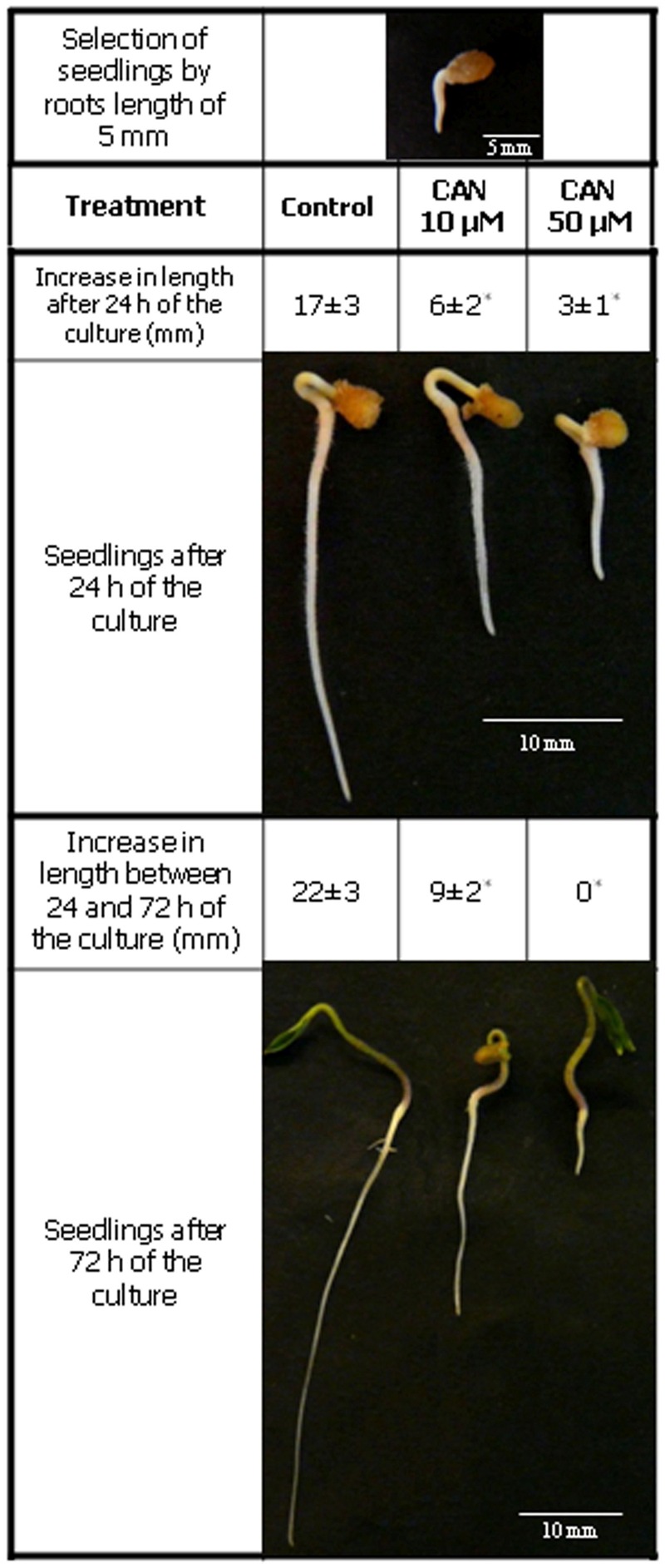
**Tomato seedlings morphology.** Equal of size, germinated seeds of tomato, with roots of 5 mm long were grown in water (control) and 10 or 50 μM CAN solution for 72 h. Increase in length of root was measured twice during the culture period: 24 h after seedlings transfer to CAN and then after additional 48 h of experiment. Values are average ± SE of five independent experiments. Asterisk (^∗^) indicate significance from control at the same time of culture period at *p* ≤ 0.05 based on Student’s test.

Seeds of tomato (*Solanum lycopersicum* L. cv. Malinowy Ożarowski) were germinated in water at 20°C in darkness for 3 days. After this period seedlings of equal roots’ length (5 mm) were separated and transferred to Petri dishes (Ø15 cm) containing filter paper moistened with distilled water (control) or CAN (Sigma-Aldrich) dissolved in distilled water pH 7.5 (**Figure 1**). Control seedlings and seedlings treated with CAN were cultured in a growth chamber at 23/20°C, 12/12 h day/night regime for 24 or 72 h.

The concentration of CAN (10 μM) required for reduction of root length to 50% of the control, was accepted as IC_50_. The concentration (50 μM), which completely inhibited elongation growth of roots, was accepted as IC_100_.

### Measurement of Hydrogen Peroxide (H_2_O_2_) Concentration

The concentration of H_2_O_2_ in extracts of tomato roots was determined according to Velikova et al. (2000). Isolated roots (100 mg) were immediately homogenized by pestle and mortar in cooled 0.1% (w/v) trichloroacetic acid (TCA). After centrifugation at 15,000 *g* for 15 min at 4°C, the supernatant was collected for further analysis. The concentration of H_2_O_2_ was measured in the assay mixture (0.25 ml supernatant, 1 ml freshly prepared 1 M KI in 10 mM potassium phosphate buffer pH 7.0, and 0.5 ml 10 mM potassium phosphate buffer pH 7.0) at 390 nm using a Hitachi U-2900 spectrophotometer. The standard curve was prepared using 0.88 μM H_2_O_2_ (Sigma-Aldrich). Measurements of H_2_O_2_ concentration were done in three independent experiments, each in three biological replicates and expressed as nmol g^-1^ FW.

Using the same method, the H_2_O_2_ concentration was measured in the liquid medium surrounding the roots. An equal number of control or CAN-treated tomato seedlings were placed in Petri dishes (Ø7.5 cm), and the culture was carried out as was described in plant material section. Then, the whole solution from the plate of the culture was collected, the volume was measured, and 0.5 ml was taken for the analyses in the reaction mixture. H_2_O_2_ concentration in the culture solution was measured at 390 nm. As a blank, water or CAN solutions from the plates without plant material were used. The experiment was done in four repetitions, and expressed as nmol L^-1^.

### Measurement of Oxidation of Epinephrine by Superoxide Radicals in Root Extract

Measurement of oxidation of epinephrine by superoxide radicals was done according to Misra and Fridovich (1972). Roots of tomato seedlings (60 mg) were homogenized in 0.05 M Tris-HCl pH 7.5 with addition of 2% (w/v) polyvinylpolypyrrolidone (PVPP), and centrifuged at 12,000 *g* for 15 min at 4°C. The supernatant was immediately used for further analyses. The oxidation of epinephrine to adrenochrome was measured in reaction mixture (0.05 ml of supernatant, 0.05 ml 0.05 M Tris-HCl pH 7.5, 0.05 ml of 60 mM epinephrine prepared in 50 mM HCl) at 480 nm (microplate reader Sunrise, Tecan) for 2 min. Autooxidation of epinephrine in the reaction mixture (without roots extract) was done in each assay and obtained values were included in the calculations. The epinephrine extinction coefficient was 𝜀 = 4.02 mM^-1^ cm^-1^. The measurements were done in three independent experiments, each in three biological replicates and expressed as relative units. One relative unit corresponds to the rate of epinephrine oxidation in root extracts of control tomato seedlings after 24 h of culture calculated as μmol min^-1^ g^-1^ FW.

### Measurement of Fluorescence Emission Corresponding to NO and ONOO^-^ Level in Tomato Roots

Nitric oxide and ONOO^-^ generation was measured as eﬄux of derivatives of 4-amino-5-methylamino-2′,7′-difluorofluorescein diacetate (DAF-FM DA, Invitrogen) and 3′-(*p*-aminophenyl) fluorescein (APF, Invitrogen), respectively following the manufactere’s instructions. APF is sensitive to ONOO^-^ but also to OH^•^, and hypochlorite (Halliwell and Whiteman, 2004).

For NO analyses, roots of three plants were washed twice in distilled water and incubated for 40 min in darkness at room temperature in the mixture: 20 μM DAF-FM DA [2 mM DAF-FM DA prepared as stock solution dissolved in dimethyl sulfoxide (DMSO)] in 10 mM HEPES-KOH, pH 7.4. After incubation, roots were washed twice in 10 mM HEPES-KOH pH 7.4 and transferred to a cuvette containing 2 ml of this buffer. Fluorescence was recorded for 1,500 s (excitation 495 nm, emission 515 nm) using a Hitachi F-2500 spectrofluorimeter. A final intensity of fluorescence in the buffer above the roots, after the indicated time was taken into calculations. After measurement the roots were gently dried on filter paper and weighed.

For ONOO^-^ determination, two roots were washed twice in distilled water and then incubated for 1 h in darkness at room temperature with 0.2% (v/v) APF [5 mM APF – stock solution dissolved in dimethylformamide (DMF)] in 10 mM HEPES-KOH, pH 7.2. After incubation roots were washed twice in 10 mM HEPES-KOH, pH 7.2 and transferred to a cuvette containing 2 ml of this buffer. Fluorescence was recorded for 1,800 s (excitation 490 nm, emission 515 nm) using a Hitachi F-2500 spectrofluorimeter. A final intensity of fluorescence in the buffer above the roots, after the indicated time, was taken into calculations. After measurement the roots were gently dried on filter paper and weighed.

Fluorescence was calculated per 1 g FW and expressed in arbitrary units (U). 1 U was estimated from the result obtained for roots isolated before treatment with CAN.

Measurements were done in 3–4 independent experiments with three repetitions in each.

### Quantitative Measurement of Protein Carbonyl Groups

Protein carbonyl groups in root extracts were measured using enzyme-linked immunosorbent assay (ELISA) according to Levine et al. (1994) and Buss et al. (1997). Roots (200 mg) were homogenized in 0.1 M Tris-HCl, pH 7.0 with 1 mM ethylenediaminetetraacetic acid (EDTA), 2% (w/v) PVPP, 1 mM dithiothreitol (DTT), 1% (v/v) protease inhibitor cocktail (Sigma-Aldrich P9599), and 10% (v/v) glycerol in an ice bath. After centrifugation at 15,000 *g* 15 min at 4°C, the supernatant was collected and incubated with 1% (w/v) streptomycin (for 20 min, at room temperature, in darkness with constant slow shaking). Then, supernatant was incubated with 10 mM 2,4-dinitrophenylhydrazine (DNPH; Sigma-Aldrich) dissolved in DMSO in the dark for 35 min at 37°C. At the same time, samples prepared without DNPH were incubated in 2 M HCl. Proteins were precipitated 10 min with 10% (w/v) TCA, and the pellets obtained after centrifugation (10,000 *g*; 10 min) were washed three times with 1:1 (v/v) ethanol:ethyl acetate. After each washing step, the samples were centrifuged for 5 min at 10,000 *g*. Washed pellets were dissolved in 6 M guanidine hydrochloride (Sigma) in 2 M HCl. Protein concentration was measured according to Bradford (1976) using BSA as a standard. Reduction of BSA fatty acid free (Sigma-Aldrich) was done using sodium dithionite (2 mM) for 30 min at 25°C in darkness. Solution of reduced BSA was passed through Sephadex G-25 column equilibrated with 0.1 M Tris-HCl, pH 7.0. Oxidation of reduced BSA was done using 1 μl of 3% (v/v) H_2_O_2_ with the addition of sodium perchlorate for 30 min at room temperature in darkness. Carbonyl groups in oxidized BSA were labeled with DNPH and processed as described above. Blank probes of BSA were incubated in 2 M HCl without addition of DNPH. Final BSA concentration was determined using Bradford reagent. Concentration of carbonyl groups in BSA was measured at 375 nm, and calculated from the extinction coefficient 𝜀 = 22 mM^-1^ cm^-1^.

Before measurement, the pH of the protein samples and oxidized BSA was adjusted to 9.0 with 5 M KOH. Triplicate of 200 μl of each sample or oxidized BSA were added to wells of Nunc Immuno Plate Maxisorp (Sigma), and incubated at 4°C overnight. Next day the samples were removed and plates were washed three times with Tris-buffered saline (TBS) with 0.05% (v/v) Tween-20 (TBST). Next, the plate wells were blocked with 0.1% (w/v) BSA in TBS (250 μl per well) for 1.5 h at room temperature. After washing step (three times with TBST), monoclonal primary antibodies (Monoclonal Anti-dinitrophenyl (DNP) antibodies, A2831 Sigma-Aldrich) conjugated with alkaline phosphatase (dilution 1:25,000) in TBST were added, at room temperature for 1.5 h in darkness. After removal of antibodies and another washing step (three times with TBST) *p*-nitrophenylphosphate disodium (pNPP), prepared at concentration 1 mg ml^-1^ in 1 M diethanolamine (DEA) solution with 0.5 mM MgCl_2_ was added into each well. Incubation of the plates were carried out at 37°C for 1 h, the reaction was stopped with 5 μl of 5 M KOH, and absorbance was read at 405 nm with referential wave 605 nm in microplate reader (Sunrise, Tecan). Measurement of protein carbonyl groups were done in three biological repetitions, each in three replicates and expressed as nmol mg^-1^ protein.

### Immunodetection of Carbonyl Groups

Immunodetection of protein carbonyl groups was done by immunoblotting technique. Protein samples prepared as described above were suspended in the sample buffer: 63 mM Tris-HCl, pH 6.8, 1% (w/v) sodium dodecyl sulfate (SDS), 10% (v/v) glycerol, 0.01% (w/v) bromophenol blue, and 20 mM DTT. After incubation at 95°C for 10 min, 20 μg proteins were loaded per lane, and separated on 10% polyacrylamide gels with SDS (SDS-PAGE) according to Laemmli (1970), and then electrotransferred to nitrocellulose membranes (Pure Nitrocellulose Membrane, Sigma-Aldrich) according to Towbin et al. (1979) using a Bio-Rad wet blotting apparatus. Subsequently, the proteins were visualized on the membranes by incubation in 0.2% Ponceau S dissolved in 2% acetic acid. The membranes were blocked overnight at 4°C with non-fat dry milk in TBST. After blocking, membranes were washed three times in TBST, and immunolabeling of carbonyl groups was carried out by incubating the membranes with monoclonal anti-DNP antibodies, conjugated with alkaline phosphatase at a dilution of 1:100,000 at room temperature. Visualization of carbonylated proteins was done after addition of 0.1 M Tris-HCl pH 9.5, 0.1 M NaCl, 5 mM MgCl_2_, 0.2 mM nitroblue tetrazolium (NBT), and 0.21 mM 5-bromo-4-chloro-3-indolyl phosphate (BCIP). Assays were performed in 2–3 independent experiments and typical results are shown. The entire gel lanes were quantified by densitometry analysis, which was done using Image J.

### Quantitative Measurement of 3-Nitrotyrosine in Proteins

3-NT modified proteins were analyzed by an ELISA method according to Khan et al. (1998). Roots of tomato seedling (200 mg) were washed twice in distilled water and homogenized by mortar and pestle in 0.1 M HEPES-KOH, pH 7.0 with 1 mM EDTA, 2% (w/v) PVPP, 2 mM DTT, 1% (v/v) protease inhibitor cocktail (Sigma-Aldrich) and 10% (v/v) glycerol in an ice bath. Following centrifugation at 15,000 *g* for 15 min, 4°C, the supernatant was collected for protein determination.

Positive control was prepared from fatty acid free BSA dissolved in TBS. BSA was incubated with NaNO_2_ (1 mM) acidified with 0.2 M HCl, and in the presence of 0.1 mM NaHCO_3_ for 30 min at 37°C, in darkness. After incubation, BSA was precipitated with 20% (w/v) TCA for 20 min at room temperature, centrifuged (10,000 *g*; 10 min), and dissolved in water adjusted to pH 9.0 with 1 M KOH. The 3-NT content of nitro-BSA was determined at 438 nm, and calculated from the extinction coefficient 𝜀 = 4.3 mM^-1^ cm^-1^.

Root protein samples before ELISA measurement were adjusted to pH 9.0 with 1 M KOH. Nunc Immuno Plate Maxisorp (Sigma) was coated with samples or nitrated-BSA (200 μl per well), and incubated at 4°C overnight. After incubation, the plate was washed three times with PBS. Following blocking step with 0.1% (w/v) gelatin in TBS (250 μl per well; 1.5 h at 37°C in darkness) and washing three times with TBST, monoclonal primary antibodies (Monoclonal Anti-3-Nitrotyrosine antibodies, Sigma-Aldrich) were added at a dilution of 1:1,000 (200 μl per well), and incubated for 1 h at 37°C in darkness. After washing three times with TBST, the plate was covered with secondary antibodies (anti-mouse IgG conjugated with Alcaline Phosphatase Sigma-Aldrich) at a dilution of 1:30,000 for 1 h at 37°C in darkness. Visualization of nitrated groups was carried out using alkaline phosphatase substrate – pNPP, prepared as described for carbonylated protein quantitative measurement. After color development, the reaction was stopped with 5 μl of 5 M KOH and absorbance was read at 405 nm with reference wavelength 605 nm in a microplate reader (Sunrise, Tecan). Measurement of 3-NT proteins were done in three biological replicates, each in three technical replicates and expressed as nmol mg^-1^ protein.

### Immunodetection of 3-Nitrotyrosine Proteins

Immunoblotting technique was done for detection of 3-nitrotyrosine proteins. Isolated proteins (as described above) were mixed with sample buffer (described above). After heating at 80°C for 5 min, samples were loaded (soluble protein – amount 25 μg per lane) and SDS-PAGE separated on 10% gels according to Laemmli (1970). An electrotrasfer of proteins to nitrocellulose membrane was done as described for carbonylated proteins. After transfer, proteins were visualized by Ponceau S Red staining. Blocking step was done using 0.1% gelatin in TBST overnight at 4°C. After the washing step nitrocellulose membranes were incubated (1 h, at room temperature in darkness) with monoclonal primary antibodies (Monoclonal Anti-3-Nitrotyrosine antibodies, Sigma-Aldrich; 1:1,000). After washing in TBST (three times), nitrocellulose membranes were covered with secondary antibodies (anti-mouse IgG conjugated with Alkaline Phosphatase Sigma-Aldrich) diluted 1:30,000 for 1 h at room temperature in darkness. Visualization of nitrated groups was carried out as described for carbonylated proteins. Assays were performed in three independent experiments and typical results are shown. The entire gel lanes were quantified by densitometry analysis, which was done using Image J.

### POx Activity Measurement

Peroxidase activity measurement was done according to Saunders et al. (1964). Roots of tomato seedling (60 mg) were washed in distilled water, and then homogenized by pestle and mortar in 0.05 M potassium phosphate, pH 7.0 with 10% (v/v) glycerol, 5 mM DTT, 1% (v/v) protease inhibitor cocktail (Sigma-Aldrich), and 2% (w/v) PVPP in an ice bath. After centrifugation (12,000 *g* for 10 min at 4°C) supernatant was collected for further analyses. Protein extract was incubated with 5 mM pyrogallol in 0.05 M potassium phosphate pH 7.0 at 25°C in darkness. After incubation, POx activity was measured after addition of 1 mM H_2_O_2_. POx activity was determined as absorbance increase at 430 nm using microplate reader (Sunrise, Tecan), and absorption coefficient 𝜀 = 2.47 mM^-1^ cm^-1^. Results were expressed as nmol H_2_O_2_ min^-1^ mg^-1^ protein. Experiments were done in at least three biological replicates, each in three technical replicates.

### PAO Activity Measurement

Polyamine oxidase activity was measured as described by Luhová et al. (2003) with some modifications. Roots of tomato seedlings (60 mg) after washing in distilled water were homogenized by mortar and pestle in extraction mixture containing 0.05 M potassium-phosphate, pH 6.5, 1% (v/v) protease inhibitor cocktail (Sigma-Aldrich), 10% (v/v) glycerol, 5 mM DTT, and 2% (w/v) PVPP in an ice bath. The homogenate was centrifuged at 12,000 *g* for 10 min at 4°C. The supernatant was collected for further analyses. PAO activity was measured in 1 ml of reaction mixture containing 0.05 M potassium-phosphate, pH 6.5, 0.5 mM guaiacol, and 1 U of horseradish peroxidase (Sigma-Aldrich) in the presence of 10 mM spermine (Spm). Activity was detected as absorbance increase at 436 nm and calculated using an absorption coefficient 𝜀 = 25.5 mM^-1^ cm^-1^. The results were expressed as nmol H_2_O_2_ min^-1^ mg^-1^ protein. Experiments were done in four biological replicates, each in three technical replicates.

### NADPH Oxidase Activity Measurement

Measurement of NADPH oxidase activity in extracts of tomato roots was done according to Paclet et al. (2007). Tomato roots were isolated, washed in distilled water and homogenized by mortar and pestle in 0.05 M Tris-HCl, pH 7.5 with 0.06 mM Tween-20, 10% (v/v) glycerol, 5 mM DTT, 1% (v/v) protease inhibitor cocktail (Sigma-Aldrich), 2% (w/v) PVPP in an ice bath. Homogenates were centrifuged (12,000 *g* for 15 min at 4°C), and supernatants were collected for further analyzes. NADPH oxidase activity was determined by reduction of cytochrome *c*. The reaction mixture contained: supernatant of 50 μg of soluble protein, 0.05 M Tris-HCl, pH 7.5, 60 mM cytochrome *c*, 1 mM CaCl_2_, 0.1 mM MgCl_2_, 0.1 mM NaCl, 0.06 mM Tween-20. In parallel, cytochrome *c* reduction was done in reaction mixture with addition of 50 U of superoxide dismutase (SOD; Sigma-Aldrich). After incubation at 30°C for 5 min (when $O_{2}^{•-}$ production from endogenous reductants had ceased) the reaction was started by addition of 0.1 mM reduced β-nicotinamide adenine dinucleotide phosphate (β-NADPH) and cytochrome *c* reduction was measured at 550 nm (Sunrise, Tecan). The presented values were calculated from differences between cytochrome *c* reduction rates in the presence and absence of SOD (Sigma-Aldrich) using an absorption coefficient 𝜀 = 21.1 mM^-1^ cm^-1^. A blank (reaction mixture without supernatant) was also done to every measurement.

The results were expressed as nmol min^-1^ mg^-1^ protein. Experiments were done in four biological replicates in three technical replicates.

### Measurement of Proteolytic Activity

Determination of proteolytic activity was done according to Pande et al. (2006) with some modifications, using azocasein as a unspecific substrate. After isolation, roots of tomato seedlings (60 mg) were washed in distilled water and homogenized by mortar and pestle in 0.1 M potassium-phosphate, pH 7.0, 0.1% (v/v) Triton X-100, 5 mM DTT, 2% (w/v) PVPP in an ice bath. After two times centrifugation (10,000 *g*, for 5 min, at 4°C), the supernatant was collected, and the protein concentration was measured using Bradford (1976) reagent. Protease activity was measured at pH 5.4 and 8.8. Supernatant containing 20 μg of total soluble proteins was pre-incubated 5 min at 37°C in 0.1 M Tris-HCl, pH 5.4 or 8.8. Subsequently 0.8% (w/v) azocasein solution was added and incubated for 30 min at 37°C in darkness. The reaction was stopped by the addition of 10% (w/v) TCA. Samples were centrifuged (12,000 *g*, for 15 min), supernatant was collected and mixed with 1 M NaOH in 1:1 ratio. After 5 min incubation, the absorbance was measured at 440 nm (Sunrise, Tecan). The specific activity of proteases was expressed as enzyme units per mg protein. Units of enzyme activity were calculated from the absorbance value increase obtained after unspecific protease activity measured in buffers of different pH at 37°C for 30 min as described above. Measurements of proteolytic activity were done in four independent experiments, each in three replicates.

### Statistics

Mean values were computed for each experiment and mean differences were calculated using analysis of variance (ANOVA) and Duncan’s test. Standard deviation (SE) are also provided to indicate the variations associated with the particular mean values. Calculations were performed using the package agricolae (de Mendiburu, 2010) for the statistical freeware R version 2.14.2 (R Development Core Team, 2012). For growth parameters data were analyzed using the StatGraphics 5.1; Mean ± SE were computed for each experiment and significance of differences was assessed with Student’s *t*-test. Differences are considered significant at *p* < 0.05.

## Results

### CAN Inhibited Elongation Growth of Tomato Roots

Treatment of tomato seedlings with CAN resulted in drastic inhibition of root growth. Inhibition of root growth by 50% was observed in CAN at concentration 10 μM (**Figure 1**), while 50 μM CAN stopped tomato root elongation completely after 72 h (**Figure 1**). Thus, 50 μM CAN was selected as IC_100_ while 10 μM as IC _50_ and used for further investigations.

### CAN Enhanced H_2_O_2_ Concentration and Superoxide Radicals Generation in Roots Extracts of Tomato Plants and Slightly Increased H_2_O_2_ Eﬄux from the Roots

Detection of superoxide radicals indicated its stable generation in roots extracts of control seedlings during the culture period (**Table 1**). In extracts of roots of control plants after 24 and 72 h of culture production of superoxide radicals was 1.125 and 1.110 μmol min^-1^ mg^-1^ FW, respectively. In roots extracts of plants treated for 24 h with 50 μM CAN generation of superoxide radicals was similar to the control, while in extracts of plants exposed to 10 μM CAN superoxide radicals generation was twice as high as in the control. Prolonged (for additional 48 h) exposure of seedlings to 10 μM CAN resulted in decline of superoxide radicals generation in roots extracts, but it was still higher than in extracts of seedlings growing in water. High generation of superoxide radicals was detected also in roots extracts of seedlings treated with 50 μM CAN for 72 h, it was twice as high as in the control (**Table 1**).

**Table 1:** Superoxide radicals generation, H_2_O_2_ concentration in root extracts and H_2_O_2_ concentration in culture medium of control tomato seedlings and seedlings treated with CAN (10 or 50 μM) for 24 or 72 h.

Plant treatment	Superoxide radicals generation (relative unit)		H_2_O_2_ (nmol g^-1^ FW)		H_2_O_2_ in culture medium (nM)	
	24 h	72 h	24 h	72 h	24 h	72 h
Control (water)	1.00 ± 0.12c	0.99 ± 0.12c	75.00 ± 5.5c	79.0 ± 6.0c	1.5 ± 0.3c	1.1 ± 0.1c
CAN 10 μM	1.94 ± 0.28a	1.26 ± 0.10b	99.5 ± 10.0b	118.0 ± 12.5a	3.6 ± 0.6a	2.7 ± 0.5ab
CAN 50 μM	1.06 ± 0.22bc	1.89 ± 0.20a	136.5 ± 13.5a	92.0 ± 8.0b	3.4 ± 0.7a	2.3 ± 0.2b

*In determination of H_*2*_O_*2*_ concentration in culture medium water or CAN solution of appropriate concentration was used as a blank. One relative unit describing the rate of superoxide radicals generation corresponds to superoxide radicals generation in roots extracts of control plants after 24 h, equal to 1.125 μmol min^-1^ g^-1^ FW. Mean of values are statistically different at *p* < 0.05, when they share no common letter(s). The comparison were made using the Duncan test. Values are average ± SE of 3–4 independent experiments.*

In roots extracts of control plants H_2_O_2_ concentration did not differ as culture period was prolonged, and was 75–80 pmol mg^-1^ FW (**Table 1**), equivalent to 78–83 μM. CAN treatment increased H_2_O_2_ level in roots extracts, leading to a doubling (about 145 μM) after application at higher concentration for 24 h. Longer (72 h) exposure of seedlings to 10 μM CAN enhanced H_2_O_2_ concentration in roots extracts to around 150% of the control (122 μM). No such effect after 72 h was observed in case of 50 μM CAN. In roots extracts of seedlings treated with 50 μM CAN, H_2_O_2_ concentration was about 116% of the control (**Table 1**), equivalent to 98 μM.

The concentration of H_2_O_2_ in the culture medium solution was very low and did not exceed 3.6 nM independently of the treatment. For control plants, during the whole experiment it was stable, and below 1.5 nM (**Table 1**). Expressed per root fresh weigh H_2_O_2_ content in the medium was significantly higher for plants treated with CAN, and after 24 h reached 461 or 547 pmol g^-1^ FW for 10 or 50 μM CAN respectively, in comparison to control (156 pmol g^-1^ FW). After 72 h culture it declined to 200 or 380 pmol g^-1^ FW for 10 or 50 μM CAN respectively, and 76 pmol g^-1^ FW in control (data not shown).

### CAN Inhibited Fluorescence of DAF-FM Derivatives Corresponding to NO Generation and Transiently Enhanced Fluorescence of APF Derivatives Corresponding to ONOO^-^ Production in Tomato Roots

In roots of control plants growing in water fluorescence of DAF-FM declined slightly (about 25%) during prolonged culture. As expected, CAN inhibited fluorescence of DAF-FM derivatives independently of concentration (**Table 2**). After 24 h fluorescence of DAF-FM derivatives had decreased to 25%, and remained at that level after 72 h.

**Table 2:** Fluorescence of DAF-FM derivatives (corresponding to NO) and fluorescence of APF derivatives (corresponding to ONOO^-^) for roots of tomato seedlings growing in water (control) or roots of seedlings treated with CAN solution at 10 and 50 μM concentration for 24 or 72 h.

Plant treatment	NO (arbitrary units)		ONOO^-^ (arbitrary units)	
	24 h	72 h	24 h	72 h
Control (water)	0.80 ± 0.07a	0.58 ± 0.06b	0.29 ± 0.06b	0.25 ± 0.06b
CAN 10 μM	0.17 ± 0.02c	0.19 ± 0.02c	0.38 ± 0.06a	0.27 ± 0.05b
CAN 50 μM	0.20 ± 0.02c	0.22 ± 0.03c	0.37 ± 0.07a	0.25 ± 0.05b

*Mean of values are statistically different at *p* < 0.05, when they share no common letter(s). The comparison were made using the Duncan test. Values are average ± SE of 3–4 independent experiments.*

Fluorescence excitation of APF used for determination of ONOO^-^ production from control roots did not differ as culture was prolonged. CAN treatment led to slight (30%) and transient enhancement in APF fluorescence after 24 h of exposure (**Table 2**). After 72 h of treatment no differences in fluorescence of APF derivatives for roots of CAN treated seedlings and control were detected.

### CAN Treatment Led to Alterations in Concentration of Protein Carbonyl Groups and the Pattern of Carbonylated Soluble Proteins

The level of protein carbonyl groups in homogenates of roots of control plants increased from 30 to 79 nmol mg^-1^ protein, as the culture period was extended for 3 days (**Table 3**). Short term (24 h) exposure to 10 or 50 μM CAN resulted in an increase of the level of carbonyl groups in root homogenates by 53 and 70%, respectively. As CAN treatment was prolonged, the concentration of stable DNP derivatives of the carbonyl groups in proteins isolated from roots increased to 104 nmol mg^-1^ protein in 10 μM CAN or 88 nmol mg^-1^ protein in 50 μM CAN (**Table 3**). In 50 μM CAN it was at the level of the control, while for 10 μM CAN it was 30% higher than in the control.

**Table 3:** Content of protein carbonyl groups and 3-NT in roots of control tomato seedlings and seedlings treated with CAN (10 and 50 μM) for 24 or 72 h.

Plant treatment	Carbonyl groups (nmol mg^-1^ protein)		3-NT (nmol mg^-1^ protein)	
	24 h	72 h	24 h	72 h
Control (water)	30.0 ± 6.5d	79.0 ± 5.0b	4.2 ± 0.1d	9.4 ± 0.2a
CAN 10 μM	46.0 ± 7.0c	104.0 ± 9.0a	5.5 ± 0.1c	6.8 ± 0.1b
CAN 50 μM	52.0 ± 9.0c	88.0 ± 6.0b	4.9 ± 0.2c	7.0 ± 0.2b

*Mean of values are statistically different at *p* < 0.05, when they share no common letter(s). The comparison were made using the Duncan test. Values are average ± SE of at least three independent experiments.*

**FIGURE 2:**
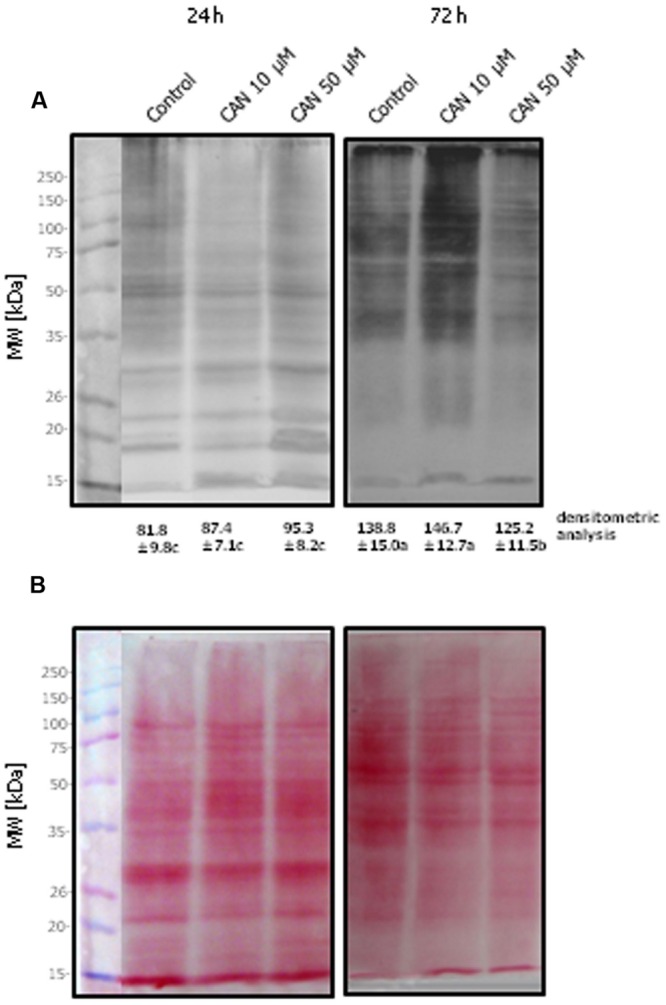
**Detection of DNPH-derivatized proteins of tomato roots isolated after 24 or 72 h of culture in water (control) or 10 or 50 μM CAN solution.** Total soluble proteins (20 μg per lane) were separated by 10% SDS-PAGE and transferred onto a nitrocellulose membrane. **(A)** Oxidized proteins were recognized by anti-DNP antibody. **(B)** The membrane was stained with Ponceau S for examination of protein transfer. The approximate molecular mass for each protein band is indicated in the panels on the left. Experiments were performed three times and representative data are shown. Mean of values of densitometric data are statistically different at *p* < 0.05, when they share no common letter(s). The comparison were made using the Duncan test.

Detection of total soluble carbonylated proteins isolated from roots of control tomato seedlings separated by SDS-PAGE (**Figure 2A**) and transferred onto a nitrocellulose membrane stained with Ponceau S (**Figure 2B**) for protein transfer examination showed significant modification in their pattern. In the protein extracts from roots treated with 10 μM CAN for 72 h many carbonylated proteins of molecular weight above 35 kDa were detected. After 72 h of culture thick bands of carbonylated proteins were detected at the top of the membrane corresponding to the top of the gel, both for the control and treated plants (**Figure 2A**). Bands of carbonylated proteins of high density were marked for the control and particularly CAN (50 μM) stressed roots after 72 h, and they were characteristic for proteins corresponding to the band of the molecular marker of 15 kDa. Ponceau S staining of the membrane after protein transfer is shown in **Figure 2B**. In roots of seedlings after 24 h treatment proteins of molecular weight of around 30 kDa were visible, but they were below detection range after 72 h. In contrast more proteins of molecular weight 35–50 kDa were detected in roots of seedlings cultured for 72 h (**Figure 2B**).

### CAN Treatment Led to a Slight Decline in Concentration of Total 3-NT and Modification in the Pattern of Nitrated Proteins

For control plants the level of nitrated proteins increased with root age (**Table 3**). It doubled from the value around 4–9 nmol mg^-1^ protein, as the culture period was extended up to 72 h. Short term (24 h) CAN treatment resulted in slight (15–30%) increase in 3-NT concentration in root proteins, as compared to plants growing in water (**Table 3**). As CAN treatment was prolonged, 3-NT concentration increased to 7 nmol mg^-1^ protein, but was 25% lower than that detected in control roots (**Table 3**).

**FIGURE 3:**
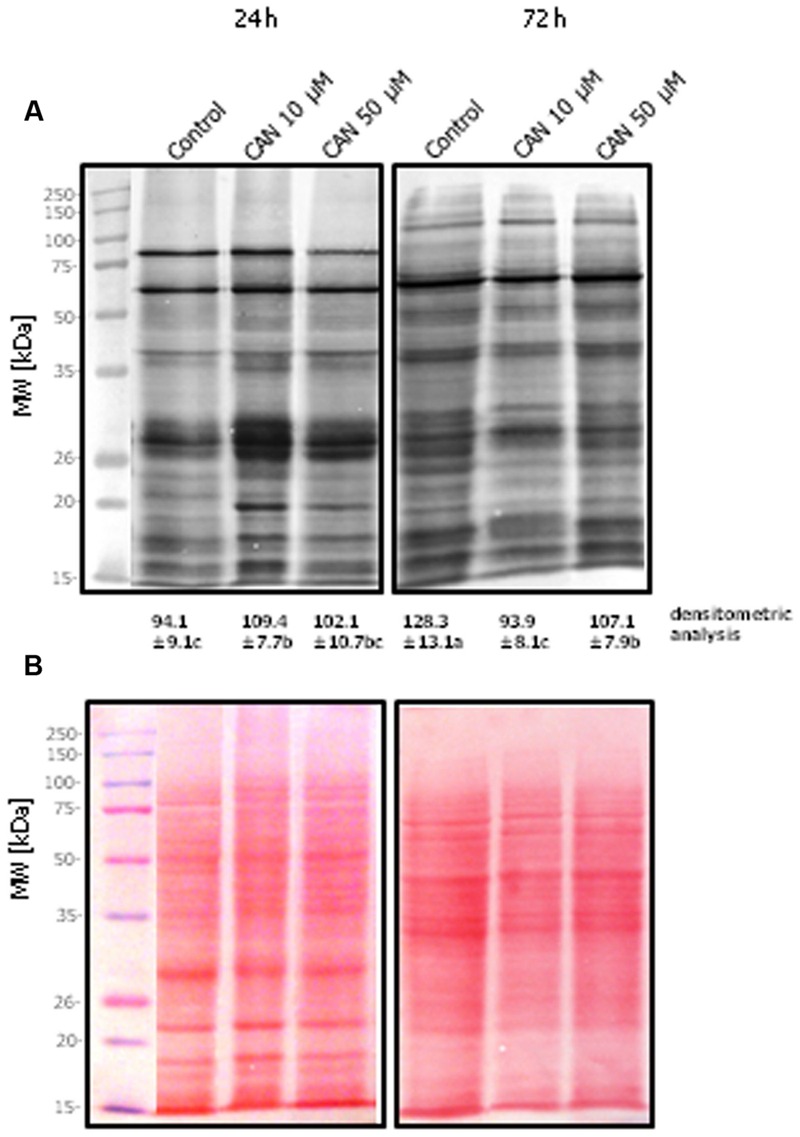
**Pattern of 3-NT modified proteins of tomato roots after 24 or 72 h of the culture.** Control – plants growing in water, CAN 10 μM- seedlings treated with 10 μM CAN, Can 50 μM – seedlings imbibed in 50 μM Can. Total soluble proteins (25 μg of per lane) were separated by 10% SDS-PAGE and transferred into a nitrocellulose membrane. **(A)** Nitrocellulose after Western blot transfer, followed by detection with monoclonal anti-3-NT antibodies, **(B)** Ponceau S stained nitrocellulose after protein transfer onto membrane. Molecular mass standards in kDa are indicated in the left panels. Experiments were performed 3–4 times and representative data are shown. Mean of values of densitometric data are statistically different at *p* < 0.05, when they share no common letter(s). The comparison were made using the Duncan test.

**Figures 3A,B** shows the protein patterns analyzed using Ponceau S staining and the corresponding tyrosine nitration protein patterns detected with an antibody against 3-NT in roots of control tomato plant or plants exposed to CAN for 24–72 h. In general, roots showed a similar protein tyrosine nitration pattern independently of the treatment, but it differed as experiment was prolonged. Intensity of staining of protein bands below 20 kDa differed in roots of CAN treated plants after 24 h in comparison to control (**Figure 3A**). In roots of seedlings exposed to CAN after 72 h a band of nitrated proteins of 20 kDa is not visible. The nitration profile of proteins isolated from roots after 72 h of the experiment was also different, with less intense band of around 90 kDa being detected. 3-NT-immunopositive proteins of molecular weight above 100 kDa were more abundant in roots after 72 h of the culture both in control and CAN stressed plants (**Figure 3A**).

### Activities of Enzymes Involved in Regulation of ROS Level Were Stimulated by CAN

**FIGURE 4:**
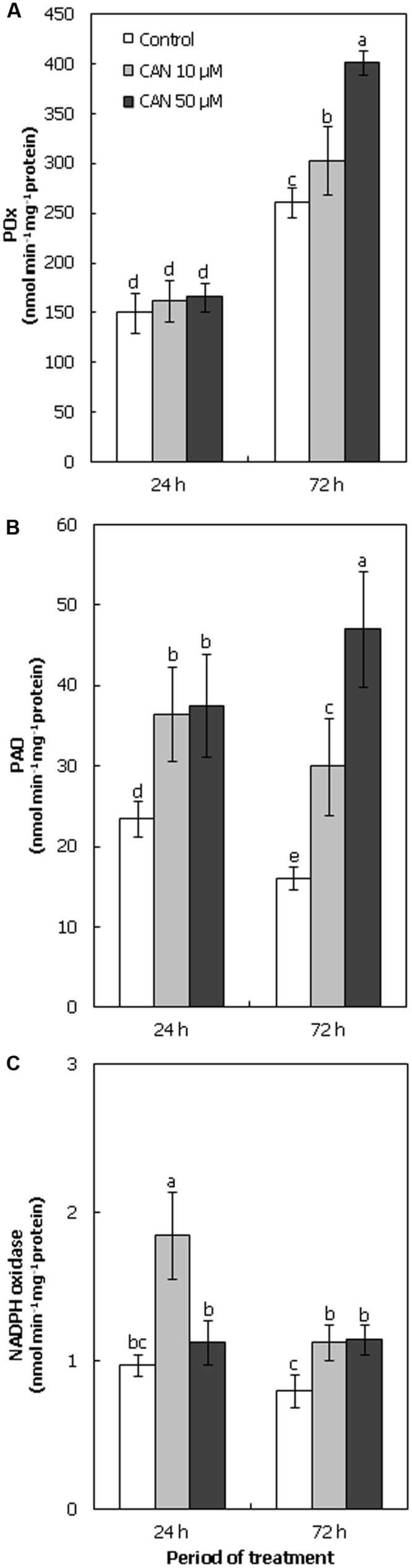
**Activity of POx **(A)**, PAO **(B)**, and NADPH oxidase (**C**; nmol min^-1^ mg^-1^ protein) in roots of tomato seedlings growing in water (control) or treated with CAN (10 or 50 μM) after 24 or 72 h of culture period.** Mean of values are statistically different at *p* < 0.05, when they share no common letter(s). The comparison were made using the Duncan test. Values are average ± SE of at least three independent experiments and three biological repetitions each.

Short-term exposure of plants to CAN did not influence POx activity in roots (**Figure 4A**). In roots of control plants and seedlings treated with CAN (10 and 50 μM) POx activity was of the same range (150–168 nmol min^-1^ mg^-1^ protein). Additional 48 h of culture resulted in enhancement of POx activity both in control and CAN stressed roots. The highest POx activity was observed in roots of seedlings treated with 50 μM CAN, and it was 160% of the control, while 10 μM CAN led to only 15% stimulation of enzyme activity (**Figure 4A**).

During the culture period PAO activity in roots of control seedlings declined from 23 to 16 nmol min^-1^ mg^-1^ protein (**Figure 4B**). CAN treatment resulted in stimulation of PAO activity, after 24 h NPAA influence was concentration independent. In roots growing in CAN for 24 h PAO activity was 60% higher than in control seedlings. Prolongation of CAN stress to 72 h led to further stimulation of enzyme activity. In roots exposed to 10 μM CAN for 72 h PAO activity was doubled as compared to control, while in roots growing in 50 μM CAN its activity was three fold higher than in non-stressed plants (**Figure 4B**).

Activity of NADPH oxidase in roots of plants growing in water was constant during the experiment (**Figure 4C**). CAN treatment stimulated activity of this enzyme. The highest activity of NADPH oxidase, twice as high as the control, was observed after short-term (24 h) application of 10 μM CAN. At 72 h its activity declined, but was still 40% higher than in the control. CAN at concentration of 50 μM led to only slight (15%) stimulation of activity of NADPH oxidase at the beginning of the experiment, but after 72 h it was about 80% higher than in plants growing in water (**Figure 4C**).

### Proteolytic Activity in Roots of Tomato Seedlings Was Enhanced by CAN Treatment

**FIGURE 5:**
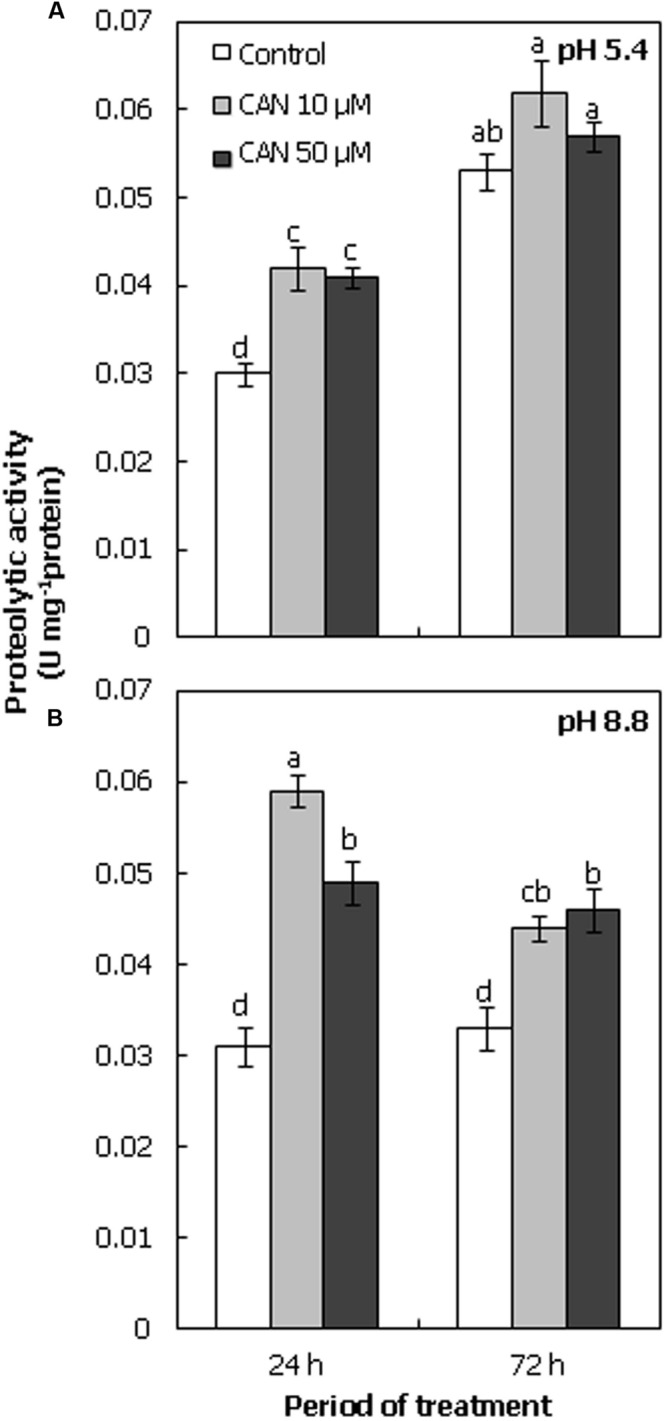
**Proteolytic activity (U mg^-1^ protein) in roots of tomato seedlings cultured for 24 or 72 h in water (control) or in 10, 50 μM CAN solution, determined at acidic pH (5.4; **A**) or at basic pH (8.8; **B**).** Mean of values are statistically different at *p* < 0.05, when they share no common letter(s). The comparison were made using the Duncan test. Mean ± SE, *n* = 10–12.

Proteolytic activity in extract of tomato roots was determined at pH 5.4 and 8.8 (**Figures 5A,B**). In roots of control plants after 24 h of growth, proteolytic activity was around 0.3 U mg^-1^ protein at both pH. After 72 h it doubled at acidic pH, and was stable at basic pH. Treatment of seedlings with CAN led to stimulation of protolytic activity at both pH. The highest proteolytic activity was observed at pH 5.4 after 72 h of culture (**Figure 5A**).

## Discussion

Canavanine is a natural NPAA produced by some plants. As a compound present in seeds or leaves it could be consumed by animals or people. Due to antiviral, antifungal, antibacterial, and anticancer properties CAN is used in medicine (Ekanayake et al., 2007). Toxicity of CAN was shown for herbivores (Rosenthal, 2001; Jang et al., 2002), but also for plants, including apple (*Malus domestica* Borkh.) embryos (Krasuska et al., 2014a). Just recently, we demonstrated that the presence of CAN in the growing medium of young (3–4 days-old) tomato seedlings led to an inhibition of root elongation growth. This effect was dose- and time-dependent (Krasuska et al., 2016). CAN is a substrate of arginyl-tRNA synthetases responsible for the incorporation of NPAA instead of Arg into a protein structure (Jang et al., 2002). Occurrence of dysfunctional proteins leads to a disruption of metabolism and finally stress induction. Common physiological reaction to abiotic or biotic stressors is ROS/RNS burst (imbalance in ROS/RNS production and scavenging; Groß et al., 2013). It has also been noticed as secondary mode of action of some allelochemicals or phytotoxins (Gniazdowska et al., 2015).

We have shown that CAN, starting from 24 h of treatment, stimulated superoxide radicals generation (**Table 1**), mostly generation of superoxide anion ($O_{2}^{•-}$), as we reported previously using root staining with NBT and correlated well to O_2_ consumption (Krasuska et al., 2016). CAN enhanced also H_2_O_2_ concentration in root extracts of tomato seedling (**Table 1**). It was accompanied by the exudation of H_2_O_2_, however, CAN did not disturb membrane permeability and roots viability (Krasuska et al., 2016). Thus, ROS exudation into the environment could be a root response to NPAA. The rise of ROS level and radicals production in root extracts of tomato seedlings exposed to CAN was comparable to those detected in *Arabidopsis* plants grown in the medium supplied with *p*-hydroxybenzoic acid (Guan et al., 2014) and to those in onion (*Allium cepa* L.) or maize (*Zea mays* L.) treated with cyanamide, an allelochemical of hairy vetch (*Vicia villosa* Roth.; Soltys et al., 2011, 2014). Enhanced $O_{2}^{•-}$ level was observed in cyanobacteria *Microcystis aeruginosa* (Kützing) after berberine application, and associated with growth inhibition (Zhang et al., 2011). Disruption of $O_{2}^{•-}$ and H_2_O_2_ ratio in root meristem resulted in restriction of root cells growth (Tsukagoshi et al., 2010). Therefore, inhibition of elongation of the tomato root by CAN could be explained by ROS-dependent disorder of cell differentiation or proliferation, which could be the secondary mode of action of this NPAA. The incubation of mouse glial cell lines with CAN resulted in an enhancement of ROS content, accompanied by a decline in the reduced form of glutathione (Riganti et al., 2003). CAN also stimulated the pentose phosphate pathway, a strong marker of oxidative stress. Moreover, in a cell-free system, CAN decreased DTT level (Riganti et al., 2003), indicating that this NPAA could take part in the regulation of redox state of small thiol containing compounds.

Canavanine lowered the fluorescence rate of DAF-FM, corresponding to NO formation in the tomato seedling roots (**Table 2**), similarly, as was detected previously for root tips (Krasuska et al., 2016). This effect seems to be uncommon, as stress factors usually induce secondary nitro-oxidative stress. On the other hand, it suggests that CAN could inhibit or disturb formation of NO *via* still unidentified Arg-dependent pathway, which seems to be important for the maintenance of regular root growth. In our experiment, a slight increase in fluorescence of APF, corresponding to ONOO^-^ production in tomato roots was observed only after 24 h of the culture in CAN solution (**Table 2**) and matches well to microscopic localization of ONOO^-^ in root tip cells (Krasuska et al., 2016). As compared to short-term CAN treatment, prolonged (72 h) exposure to CAN resulted in a decline in the ONOO^-^ generation. The transient enhancement of ONOO^-^, measured using APF, can be linked to an elevated production of ROS (particularly OH^•^).

The increase in the content of protein carbonyl groups is a commonly accepted marker for ROS imbalance. There is no information about the impact of allelopathic compounds on the level of protein carbonyl groups in tissues of acceptor plants. The treatment of tomato seedlings with CAN led to an increased concentration of carbonyl groups (**Table 3**). In CAN treated roots higher abundance of carbonylated proteins was especially observed after 72 h (**Figure 2**). Observing a band on the membrane corresponding to the front of the gel we can conclude that CAN led to increased abundance of carbonylated proteins of low molecular weight. According to the concept of Møller and Sweetlove (2010) some carbonylated peptides released from proteins, after their breakdown, can serve as signaling molecules of specific cellular compartment origin. Previously, it was demonstrated that several storage proteins, in seeds are carbonylated to accelerate their mobilization during the germination process (Job et al., 2005; Krasuska et al., 2014b). In high abundance, we have also detected carbonylated proteins that were not entering the gel. These were probably aggregates of various proteins, content of which increased after 72 h of the culture, both in CAN treated and control plants (**Figure 2**). Protein aggregates may diminish their ability for degradation (Jung et al., 2007). The physiological explanation for the accumulation of aggregates is hard to clarify, however, such phenomenon occurs also in regularly growing roots. Differences between quantitative detection of carbonylated proteins (**Table 3**) and results of Western blot assay (**Figure 2**) were due to absorption of proteins and/or fragments of proteins on Maxisorp plates. Maxisorp plates bind also small proteins, which can be lost in protein transfer during Western blot analysis. It could be also suspected that due to high proteolytic activity after treatment with CAN (**Figure 5**) fragments of small proteins, which were bonded to the surface of the plates were lost in Western blot.

During the progression of the leaves vegetative development in *Arabidopsis* cultured under optimal conditions, an increase of the level of carbonylated proteins was detected (Johansson et al., 2004). Similarly, we observed that the alterations in the level of protein DNP derivatives in roots of the control tomato seedling, during the progression of growth, were of the same pattern as described in the roots of apple seedlings (Krasuska et al., 2014b). The content of carbonyl groups in tomato roots at the beginning of the experiment (roots were of length of 5 mm) was higher (data not shown) than in the roots of plants growing in water for additional 24 h. During the extension of root elongation growth the amount of carbonyl groups in proteins doubled (**Table 3**). Alike, in roots of apple embryos at the termination of germination *sensu stricto* higher level of carbonylated peptides, than in the roots of 10-days-old seedlings cultured in water, was noticed (Krasuska et al., 2014b). Thus, it implies that accumulation of some carbonylated proteins could be due to physiological processes occurring during growth and development, not simply related to the risks of oxidative stress.

Bearing in mind that ROS act as key molecules in the regulation of root growth and development, specific carbonylated proteins may be involved is signal transduction. There is also a possibility, that carbonylation could serve as a protective mechanism, that addresses/directs putative aberrant proteins enriched in CAN to faster degradation. Isolation and identification of such proteins would be of great interest to explain CAN toxicity. The more, in HEp-2 cells it was shown that endogenous canavanyl proteins are preferentially ubiquitinated and probably processed to degradation via proteasome (Akaogi et al., 2006).

The mode of action of RNS involves a modification of protein amino acid residues such as, e.g., Tyr (Astier and Lindermayr, 2012). Following disturbances in RNS level after CAN application we measured the 3-NT level in proteins isolated from roots of tomato seedlings (**Table 3**). Just like the content of protein carbonyl groups points at ROS metabolism, amplification of Tyr nitration can serve as a marker of nitrosative stress. CAN treatment led to a slight increase followed by a decline of 3-NT in proteins in roots of tomato seedlings (**Table 3**). The increase in the 3-NT level after short term CAN treatment is in agreement with the data obtained for ONOO^-^ production, and can also be due to the higher abundance of small proteins. The lower (in comparison to the control) level of 3-NT groups after prolonged CAN supplementation indicated that NO cellular production was limited, or NO production was unaffected, but the reaction with $O_{2}^{•-}$ was accelerated. Increase of $O_{2}^{•-}$ (free radical burst) can be also a reason of lowering cellular NO concentration, as these compounds react rapidly. On the other hand, we have demonstrated equal concentration of NO_2_^-^ in CAN stressed and non-treated tomato seedling (Krasuska et al., 2016). ONOO^-^ is not the only one nitration agent, as this process occurs also in the presence of NO_2_^-^ (Speckmann et al., 2016). Thus, we suspect that CAN influences other mechanisms of NO generation (probably enzymatic pathways), being beneficial during root growth and development.

Summing up these results with those of NO eﬄux we can conclude that CAN serves rather as inhibitor of RNS burst, and in consequence, may have a stressful effect. Therefore, the restriction of root growth in tomato seedlings by CAN could be linked to the disturbances in RNS tissue level. NO is required for an undisturbed root growth (Yu et al., 2014). Corpas et al. (2009) indicated that in roots of 71-days-old pea (*Pisum sativum* L.) plants growing under optimal conditions proteins with 3-NT were present at a level higher than in other organs, e.g., leaves or flowers. So, protein nitration is of physiological occurrence, similarly as we previously pointed to carbonylation. In 2-weeks-old seedlings of *Arabidopsis* more than 60% of the identified nitrated proteins were engaged in primary metabolism (Lozano-Juste et al., 2011), suggesting that inhibition of this PTM and/or deficiency of specific nitrated proteins could disrupt cellular homeostasis. We can also assume that peptides/proteins with 3-NT could serve as signaling molecules of specify nature and origin. Our data indicates that CAN treatment destabilizes this potent 3-NT-dependent signaling pathway. Tyr nitration increased during various stress conditions (Corpas et al., 2008). Stress factors led to a substantial strengthening of protein bands immunolabeled against 3-NT of molecular weight of 29–59 kDa isolated from pea plants. The treatment of tomato roots with CAN led to a transient (in first 24 h) increase of the intensity of 3-NT-labeling of 20 and 30 kDa protein band, while in the control samples these bands were almost at the same level up to 72 h (**Figure 3**). These proteins are probably associated with the phytotoxicity of CAN, and thus needs identification.

Canavanine treatment of tomato seedlings increased proteolytic activity (**Figure 5**). Moreover, the transient (observed after 24 h) increase in protein nitration and carbonylation (compared to the control) could favor their proteolysis. Protein nitration enhances the susceptibility to degradation by proteasome pathway, on the other hand oxidative/nitrosative stress conditions lead to an inactivation of proteasome (Souza et al., 2000; Kästle et al., 2012). Furthermore, it was shown that at high cellular ROS level oxidatively modified proteins were degraded by peroxisomal proteases (Romero-Puertas et al., 2002). In addition, proteases participate in the cellular defense system (Fowler et al., 2009), so we can conclude that a higher proteolytic activity after plant treatment with CAN is part of the detoxification machinery. Aberrant products of CAN incorporation into mitochondrial proteins of human cells lines were detected only for a short time, suggesting their enhanced degradation (Konovalova et al., 2015). Similarly, the decrease in the content of carbonylated proteins during apple embryo germination, the process stimulated by NO or hydrogen cyanide (HCN), was linked to a stimulation of proteolytic activity (Dębska et al., 2013; Krasuska et al., 2014b).

As mechanical properties of cell wall depend on ROS interaction with cell wall components, inhibition of root elongation of tomato seedlings observed after CAN treatment could be connected to the destabilization of cell wall loosening – stiffening process. We focused on the activity of ROS producing enzymes: PAO, POx, and NADPH oxidases (**Figure 4**). PAOs catalyze oxidative degradation of PAs: spermine (Spm) and spermidine (Spd), polycationic compounds belonging to the group of plant growth and development regulators. Co-product of this reaction is H_2_O_2_ (Kusano et al., 2008). Supplementation with CAN stimulated PAO activity in tomato roots, especially after 24 h of the culture (**Figure 4**), suggesting involvement of CAN in PAs metabolism. PAs take part in plant responses to various stress factors, including allelochemicals (Kusano et al., 2008; Gniazdowska et al., 2015). 2(3H)-benzoxazolinone decreased PAs content in roots of lettuce (*Lactuca sativa* L.; Sánchez-Moreiras and Reigosa, 2005). Cinamic acid reduced Spm and Spd levels in leaves of cowpea [*Vigna unguiculata* (L.) Walp.], which could be explained by the stimulation of PAO activity. Moreover, it was accompanied by an induction of oxidative stress (Huang and Bie, 2010).

Other enzymes involved in ROS metabolism are POx, especially extracellular heme-containing Class III peroxidases. The activity of POx of apoplastic localization is connected with cell wall stiffening by participation in lignin production (Liszkay et al., 2003, 2004). As was mentioned above, CAN treatment of tomato plants resulted in an inhibition of elongation growth of roots, which in addition became visibly thicker than the control. The higher POx activity in roots of seedlings treated with CAN, observed especially after 72 h of the culture (**Figure 4**), can be linked to lignin production and root stiffening. Up-regulation of the gene coding POx accompanied by an elevated level of ROS was noted in plants treated with gallic acid (Rudrappa et al., 2007; Golisz et al., 2008), which indicates that this enzyme has a function in plant response to phytotoxins. Oxygen free radicals are products of NADPH oxidases, the activity of which is coupled with the regulation of growth of root tip cells (Foreman et al., 2003), and response to allelochemicals (Oracz et al., 2012). Cinnamic acid increased ROS production, accompanied by the stimulation of NADPH oxidase activity in roots of cucumber seedlings (Ding et al., 2007). In our experiment CAN supplementation enhanced the activity of NADPH oxidases in tomato roots (**Figure 4**). These results correspond well with transiently elevated ONOO^-^ and 3-NT level in proteins in roots treated with CAN. An increase in NADPH oxidase activity pointed, that indeed $O_{2}^{•-}$ production after CAN application was greater and higher $O_{2}^{•-}$ level was not due to lower NO and its lower conversion to ONOO^-^. In contrast, the reduced expression of genes encoding NADPH oxidase in transgenic cress (*Lepidium sativum* L.) seedling resulted in inhibition of root length (Müller et al., 2012).

Canavanine disrupts vegetative growth and development of young tomato seedlings and inhibits elongation of the primary root. CAN acts as stress factor, leading to an overproduction of ROS. In addition, CAN dependent stress generation in plant tissue is also due to a decline in the cell’s RNS formation, resulting in the maintenance of RNS concentration at the level below its physiological rate. Our data indicates that the incorporation of CAN into proteins is not the only origin/base of CAN phytotoxicity. By imbalance in ROS/RNS CAN impacts protein PTMs, thus almost certainly affects the primary metabolism. Taking into account the results of the presented experiments, it would be of great importance to identify the aberrant CAN incorporated proteins, and verify their sensitivity toward redox dependent PTMs.

## Author Contributions

The experiments were conceived and designed by: UK and AG, RB helped in research discussion. The experiments were performed by: OA, PS, and UK. The data were analyzed by: UK, AG, OA, and PS. The paper was written by: UK and AG. All authors read and approved the final version of the manuscript.

## Conflict of Interest Statement

The authors declare that the research was conducted in the absence of any commercial or financial relationships that could be construed as a potential conflict of interest.

## Abbreviations

Arg: arginine
CAN: canavanine
cPTIO: 2-(4-carboxyphenyl)-4,4,5,5-tetramethylimidazoline-1-oxyl-3-oxide
3-NT: 3-nitrotyrosine
NO: nitric oxide
PTMs: post-translational protein modifications
RNS: reactive nitrogen species
ROS: reactive oxygen species
